# Imaging Time Series of Eye Tracking Data to Classify Attentional States

**DOI:** 10.3389/fnins.2021.664490

**Published:** 2021-05-28

**Authors:** Lisa-Marie Vortmann, Jannes Knychalla, Sonja Annerer-Walcher, Mathias Benedek, Felix Putze

**Affiliations:** ^1^Cognitive Systems Lab, Department of Mathematics and Computer Science, University of Bremen, Bremen, Germany; ^2^Creative Cognition Lab, Institute of Psychology, University of Graz, Graz, Austria

**Keywords:** convolutional neural network, eye tracking, classification, Imaging Time Series, Augmented Reality, Gramian Angular Fields, Markov Transition Fields, attention

## Abstract

It has been shown that conclusions about the human mental state can be drawn from eye gaze behavior by several previous studies. For this reason, eye tracking recordings are suitable as input data for attentional state classifiers. In current state-of-the-art studies, the extracted eye tracking feature set usually consists of descriptive statistics about specific eye movement characteristics (i.e., fixations, saccades, blinks, vergence, and pupil dilation). We suggest an Imaging Time Series approach for eye tracking data followed by classification using a convolutional neural net to improve the classification accuracy. We compared multiple algorithms that used the one-dimensional statistical summary feature set as input with two different implementations of the newly suggested method for three different data sets that target different aspects of attention. The results show that our two-dimensional image features with the convolutional neural net outperform the classical classifiers for most analyses, especially regarding generalization over participants and tasks. We conclude that current attentional state classifiers that are based on eye tracking can be optimized by adjusting the feature set while requiring less feature engineering and our future work will focus on a more detailed and suited investigation of this approach for other scenarios and data sets.

## 1. Introduction

Scientists' fascination for human eye gaze behavior started as early as in the 19th century when it was observed that the eyes don't move in one fluent motion while reading. Instead, they stop and focus often but only briefly. This observation led to many questions: When do they stop? Where do they focus and how long? And most importantly, why? In 1908, Edmund Huey published the first version of his book “The psychology and pedagogy of reading” (Huey, 1908) in which he discussed these observations and introduced one of the first versions of an eye tracking device. It consisted of a special contact lens that was connected to an aluminum pointer. Since then, the field of eye tracking has flourished and continuously improved eye tracking devices. In 1980, Marcel Adam Just and Patricia A. Carpenter proposed their Eye-Mind assumption, stating that “there is no appreciable lag between what is being fixated and what is being processed” (Just and Carpenter, 1980). While, this statement is restricted to eye fixations, it can be assumed that gaze behavior, in general, is closely tied to mental processes. Our knowledge about saccades and fixations, their cause and reason, and their connection to the current mental state of the observed person has increased immensely since then and the practice of eye tracking has found many applications. In addition to the mentioned research interests, human gaze tracking is widely used in consumer and marketing research (Wedel and Pieters, 2008) or as an input mechanism for technical devices, such as smartphones (Paletta et al., 2014) and Augmented and Virtual Reality glasses (Miller, 2020).

Some applications are mainly interested in the direction of the gaze (i.e., to predict salient regions of web pages as in Buscher et al., 2009). Others, however, make use of implications about the mental state that can be drawn from the eye tracking data. One famous and possibly life-saving use of eye tracking is to detect a high cognitive workload (Palinko et al., 2010), or high level of fatigue (Horng et al., 2004) in car drivers. Di Stasi et al. (2013) suggested that ocular instability increases with mental fatigue, meaning that saccadic and microsaccadic velocity decreases and drift velocity increases. If this movement behavior is observed in a driver, they can be advised to take a break from driving.

Another interesting application field for mental state classification that is gaining interest in the current Covid-19 pandemic is digital learning settings. The learning system could for example detect phases of mind-wandering. This information about the mental state of the learner can then be used to later present the corresponding content again during phases of concentration and thus, improve the chances of a better learning rate and greater learning success (Conati et al., 2013). The aspects of the human mental state that can be classified or detected are manifold. Besides the mentioned workload, fatigue, and mind-wandering, further cognitive and affective states can be modeled, such as internally and externally directed attention, attentional shifts, emotions, the direction of attention, goal-directed and task-related internal attention, or alertness.

In many studies, mental state classification is based on data from other biosignals, such as brain activity. Often, electroencephalography (EEG) is chosen for its good temporal resolution and low cost (in comparison to fMRI), as for example in Zeng et al. (2018), Dehais et al. (2018), Vézard et al. (2015), Benedek et al. (2014), Ceh et al. (2020), and Vortmann et al. (2019a). However, compared to eye tracking devices, the setup time highly depends on the number of electrodes and usually requires qualified assistance for the user. In comparison, eye tracking has the obvious advantages of a fast setup, easy calibration, and the fact that eye tracking glasses promise a better usability experience in the wild than tight EEG-caps.

The movement of the eyes is typically recorded as a time series of gaze point coordinates from both eyes. Some systems additionally record pupil diameters or blinks. Once this data is acquired, it needs to be processed so the important information can be extracted and used to draw conclusions about the mental state of the user. Typical features that are calculated on the data include the number and length of fixations, saccades, and microsaccades, the gaze velocity, the pupil size, the frequency of blinks, or the covered gaze distance. With this set of features, a supervised machine learning algorithm can learn to model the mental states of interest and detect these states in the user. One major challenge in improving the accuracy of mental state classification based on eye tracking data is finding and optimizing the right features and algorithms. In recent years, the machine learning community has solved more and more problems using deep learning approaches and neural nets because they require less feature engineering and are thus more suitable if there is a lack of domain understanding. They are used in a variety of scenarios from forecasting to fraud detection and financial services or image recognition.

Wang and Oates (2015) suggested that time series data could be represented as images or matrices (Imaging Time Series, ITS) and then these can be classified by Convolutional Neural Networks (CNN) which have proven to be successful in image classification in the past. To transform the variables from one-dimensional time series to two-dimensional images, they suggest two different algorithms: Gramian Angular Fields (GAF) which represent the temporal correlation between time points, and Markov Transition Fields (MTF) which calculate a matrix based on transition probabilities (see section 2.2.2).

In this work, we compare one-dimensional (1D) statistical summary feature set based approaches with ITS approaches for the detection of attentional states on three different eye tracking data sets related to attention. The first data set contains phases of internally and externally directed attention during several screen-based tasks (see section 2.1.1). The second data set is on the same aspect of attention but was collected in an Augmented Reality scenario (see section 2.1.2). Likewise, the third data set was collected during an Augmented Reality task but consists of phases on attention on real and phases of attention on virtual objects (see section 2.1.3). The aim is to improve the classification accuracy for multiple aspects of attention for both person-dependently and person-independently trained models. To the best of our knowledge, no previous study has performed such a comparison with the suggested methods on eye tracking data.

### 1.1. Related Work on Mental State Classification From Eye Behavior

Related studies that aimed at classifying mental states and especially attentional states from eye tracking data guided us in finding state-of-the-art features for our 1D statistical summary feature set and gave us an overview over which algorithms should be used for the comparison. Additionally, their results show that it is possible to reliably detect these states in eye tracking data.

The popular topic of eye movements during reading tasks was picked up again in a study by Faber et al. (2018) who detected phases of mind wandering based on fixations, saccades, blinks, and pupil size. They mention that these content-independent features work best for 12-s windows. Bixler and D'Mello (2016) compared the same features in a reading task with more task and content-specific features, such as repeated fixations on words. However, the general features performed better which allows for the conclusion that the general task-independent features could reach a good performance in other mind wandering and attention contexts as well. Several studies concentrated on gaining a further understanding on how fixations (Foulsham et al., 2013; Frank et al., 2015), saccades (Li et al., 2016), and eye blinks (Oh et al., 2012) are influenced by mental states. Features that were often extracted for the feature sets in the respective time interval include the number of fixations, saccades, and blinks, as well as their average length, standard deviation, median, minimum, and maximum of the length, as well as angles between saccades and the ratio of fixations and saccades. Additionally, mean, standard deviation, median, minimum, and maximum were also calculated for the pupil diameter. However, Bixler and D'Mello (2016) note that the pupil diameter is very sensitive to luminance changes in the surroundings and requires a very careful and controlled setup. Nonetheless, the connection between mental states and the pupil diameter is also assessed in the studies by Franklin et al. (2013), Pfleging et al. (2016), Unsworth and Robison (2016), and Toker and Conati (2017). Mills et al. (2016) extended the mind wandering experiments to free viewing of films and found the same results for content-independent features compared to content-dependent features. The fixation and saccade features were also used in Hutt et al. (2017) who classified mind wandering during lecture viewing using a Bayes Net. In the mentioned studies by Faber et al. (2018) and Bixler and D'Mello (2016) many different algorithms were compared to find the best performance for the feature sets. For Faber et al. (2018) the highest performance was achieved with a Logistic Regression and for Bixler and D'Mello (2016) the best results were achieved by a Bayes Net and a Naïve Bayes algorithm.

A different feature set was tested by Xuelin Huang et al. (2019) who wanted to detect internal thought from eye vergence behavior features in three different tasks (math, watching a lecture video, and a daily activity like reading or browsing the internet). They used information from two different measures: pair-based vergence features and fixation-based vergence features. Their vergence feature set was compared to a feature set containing the previously mentioned features and the performance reached a similar level or even better results. If the features were combined, the best results were achieved. A comparison of several classification algorithms showed that a random forest yields the best results. It was suggested in Puig et al. (2013) that distinguishable eye vergence features are mainly related to covert visual attention tasks. In the literature, eye vergence features were found to be related to covert visual attention (Puig et al., 2013), imagination (Laeng and Sulutvedt, 2014) and internally and externally directed cognition (Benedek et al., 2017; Annerer-Walcher et al., 2020). Hence, eye vergence features are interesting features for the classification of attentional states.

Two of the data sets that are analyzed in this work focus on the classification of internal and external attention. Internally directed attention refers to attention that is independent of stimuli from the surroundings such as memory recall or mental arithmetic. Externally directed attention instead means focusing on sensory input, for example, visual search tasks or auditory attention to one of many speakers (Chun et al., 2011). Several studies found differences in eye behavior between internally and externally directed attention, especially for various features of pupil diameter, eye vergence, blinks, saccades, microsaccades, and fixations (e.g., Salvi et al., 2015; Unsworth and Robison, 2016; Benedek et al., 2017; Annerer-Walcher et al., 2020). Some features were more consistently associated with internally and externally directed cognition than others. It is hypothesized that two mechanisms mainly lead to the differences in eye behavior between internally and externally directed attention: decoupling of eye behavior from external stimuli (Smallwood and Schooler, 2006) and coupling of eye behavior to internal representations and processes (e.g., luminance and distance, Laeng and Sulutvedt, 2014). A detailed review of the general occulometric features that were mentioned before during internal and external attention was described in Annerer-Walcher et al. (2021). In Vortmann et al. (2019b), the authors implemented a real-time system that classifies internal and external attention based on multimodal EEG and eye tracking data. For the eye tracking data they used the previously described standard features (fixations, saccades, blinks, and pupil diameter), and classified short sequences of 3 s using a Linear Discriminant Analysis (LDA). This real-time classifier was later implemented in an attention-aware smart home system to improve the usability (Vortmann and Putze, 2020).

### 1.2. Related Work on Deep Learning for Eye Tracking

In more recent advances, deep learning approaches are used to improve different areas of eye tracking. Most of these studies do not focus on differentiating mental states from the data but rather improving the gaze estimation itself, unsupervised feature extractions, or predictions about the demographics of the participants. The use cases for the applications are many-fold, such as websites (Yin et al., 2018) or Augmented and Virtual Reality (Lemley et al., 2018).

As mentioned in the previous related work, the feature engineering for eye tracking classification remains a main research area. In Lohr et al. (2020), the authors explore using a metric learning approach to extract eye gaze features. They trained a set of three multilayer perceptrons to find fixations, saccades, and post-saccadic oscillations and reached benchmark performance for the detection. However, Bautista and Naval (2020) argue that extracting features based on fixations and saccades does not represent the richness of information available in eye tracking data. They suggest using deep unsupervised learning instead of feature engineering. Two autoencoders (AE) are trained on position and velocity information to extract macro-scale and micro-scale information and fitted the representations using a linear classifier. Their classification accuracy to discriminate gender and age groups reaches competitive levels compared to supervised feature extraction methods. Zhang and Le Meur (2018), instead, classified scanpaths using a one-dimensional CNN to predict the age of the participant.

Overall, using the scanpaths in the classification process instead of extracted statistical features can be observed in several recent studies. Assens et al. (2018) and Bao and Chen (2020) predict visual scanpaths using GANs and a deep convolutional saccadic model. In Fuhl et al. (2019), the scanpaths are represented by emojis in the first step. These representations were learned by a generative adversarial network (GAN). In a second step, the emojis are classified using a Convolutional Neural Network (CNN) to predict the stimulus. The authors argue that by adding the intermediate step of the emoji representation, they increase the classification accuracy compared to classification simply based on scanpaths.

Sims and Conati (2020) used a combination of a Recurrent Neural Network (RNN) and a CNN to detect user confusion from eye tracking data. They argue that the parallel use of the neural nets allows keeping temporal information (using the RNN) and visuo-spatial information (using the CNN) and that their approach outperforms state-of-the-art classifiers. They used a 1-layer Gated Recurrent Unit (GRU) for the sequential eye tracking data and supplied the CNNs with scanpath images.

Another approach without explicit feature extraction was implemented by Zhang et al. (2019). They used a Deep Neural Network that was made up of several Long-Short-Term-Memories (LSTMs) to accurately detect Fetal Alcohol Spectrum Disorder in young children based on their natural viewing behavior.

Moving away from designated eye tracking devices, several studies have explored using other cameras for gaze detection. Different deep learning strategies have been applied in these studies to increase the tracking and classification accuracies of such systems. For example, Meng and Zhao (2017) used webcams and proposed to use five eye feature points for the tracking instead of only the iris center. These five points are detected using a CNN and afterward, another CNN is used to recognize different eye movement patterns. The iTracker by Krafka et al. (2016) is a CNN trained on a large-scale eye tracking dataset to predict gaze points without calibration based on the camera of a mobile device. It reaches state-of-the-art accuracy. CNN-based feature extraction for eye tracking using mobile devices was also assessed in Brousseau et al. (2020), where the authors suggest the combination of the camera with a 3D infrared model.

As mentioned before, Wang and Oates (2015) proposed to encode time series data as images and classify these images using CNNs. The resulting images could be a well-suited alternative to classical feature engineering for eye tracking, scanpaths, or raw data. The authors suggest two different approaches: Gramian Angular Fields and Markov Transition Fields. The two approaches are described in more detail in section 2.2.2. In their paper, they tested these two approaches as well as their combination on the twelve standard benchmark time-series datasets of language data and vital signs used in Oates et al. (2012) and compared them to state-of-the-art classifiers. The analysis showed that the new approaches reach similar results. Since then, their suggested methods have been applied in several other studies. In Thanaraj et al. (2020), the authors used the GAF successfully to classify EEG data for epilepsy diagnosis and in Bragin and Spitsyn (2019) GAF was used for motion imagery classification from EEG. We are not aware of eye tracking datasets that have been analyzed with MTF or GAF images.

## 2. Methods

Pursuing the goal of a general assessment of the usability of the imaging time-series approach for eye tracking classification of attentional states, we decided to compare multiple classifiers on multiple data sets for their classification results. The datasets cover different aspects of attention and were either recorded for screen-based tasks or in Augmented Reality. Especially Augmented Reality devices with head-mounted displays offer a good opportunity to include an eye tracker in the headset and add an explicit or implicit option for user interaction. The latest generations of Augmented Reality devices even have built-in eye tracking. Available relevant work was used as a guideline to decide on the classifiers to compare. The general occulometric features that were mentioned in section 1.1 in combination with different classifiers that we found in earlier studies will be called “Statistical Summary Approaches” (see section 2.2.1). These 1D statistical summary approaches as classification algorithms will be compared with each other as well as with two different neural nets that were trained on a feature set that was generated by the Imaging Time Series approach from Wang and Oates (2015) (see section 2.2.2). Further, we evaluate different settings for the ITS approach as well as person- and task-dependence.

### 2.1. Data Sets

The three chosen data sets are different with regard to evoked attentional focus, mode of task presentation, tasks, number of recorded participants, and total number of trials and trial lengths. They were all recorded specifically targeting a binary classification between two states of attention. Two of the data sets were recorded during experiments that were controlled for internally and externally directed attention—two modes of attention that are usually alternated unconsciously in everyday life. The third data set contains trials of only externally directed visual attention. This visual attention is either directed toward real objects or virtual objects that are displayed by an Augmented Reality device. All three experimental tasks and setups will briefly be described in the following. All experiments were approved by their local ethics committees. Please refer to the original articles for a more detailed description. An overview of the data sets can be found in Table 1.

**Table 1:** Overview of the three data sets including information about the tasks and scope.

**Data set**	**Attention**	**Task presentation**	**Participants**	**Total trials**	**Trial length (s)**
Switch	Internal/external	Screen-based	172	Approx. 15,000	10
Align	Internal/external	Augmented Reality	14	Approx. 900	15
Pairs	Real/virtual	Augmented Reality	13	Approx. 400	20

#### 2.1.1. Switch-Task

**Figure 1:**
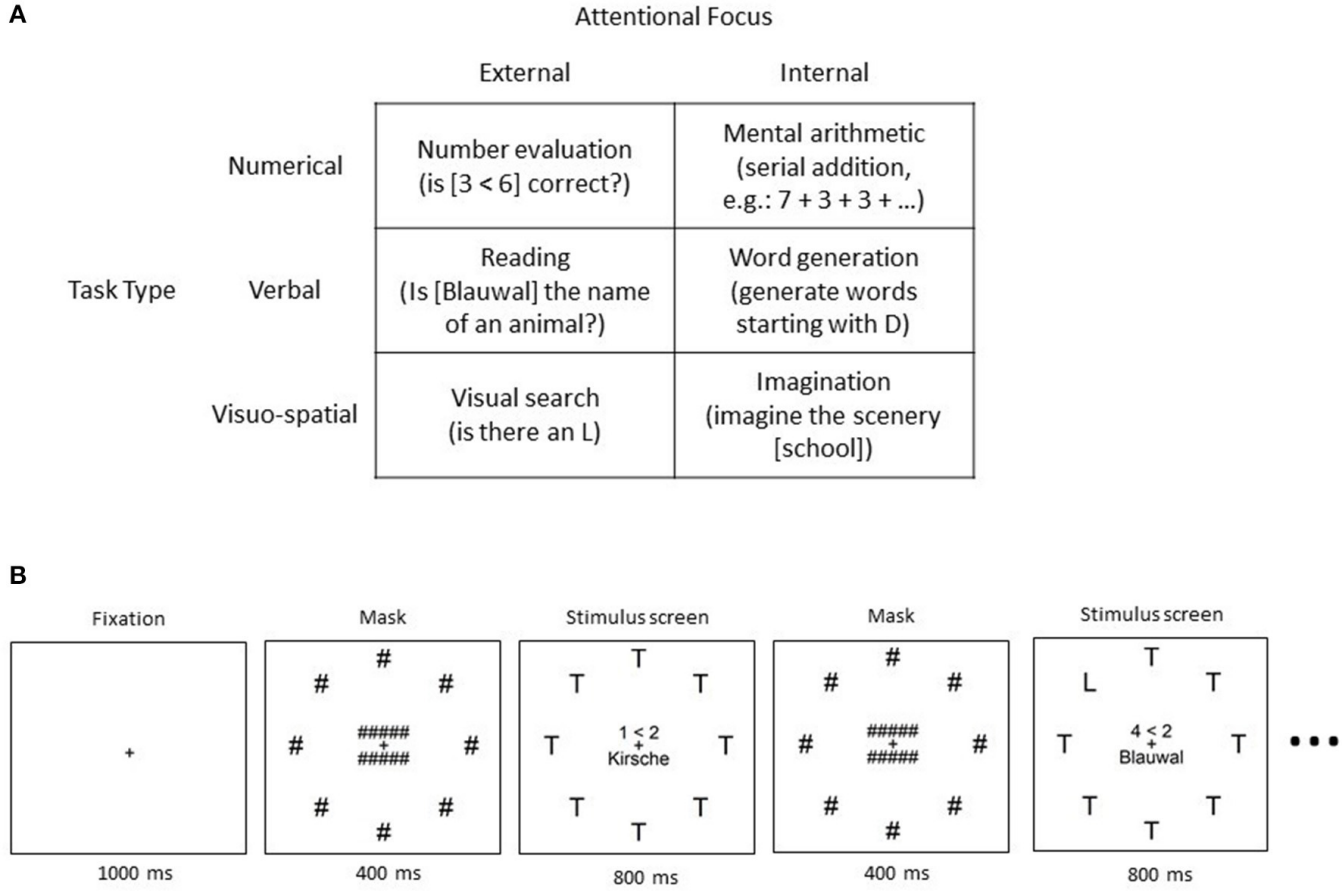
The switch-task: **(A)** Categorization of the 6 different tasks with examples for each category. **(B)** Schematic description of the procedure of one trial, including example stimuli and timing information. Taken from Annerer-Walcher et al. (2021).

The original research article of the switch-task data set was published in Annerer-Walcher et al. (2021). It was recorded as a cooperation of the University of Graz, Austria, and the University of Bremen, Germany. During the experiment, the participants were presented with 6 different types of tasks on a computer screen (see Figure 1A for task types). Each task was either numerical, verbal, or visuo-spatial and required either internally or externally directed attention. Participants were advised to keep their eyes open and focused on the screen, independent of the task. A task description was displayed before each trial. After a button press, a drift correction was performed while the participants focused on a fixation cross. For external attentional focus trials, it was necessary to attend the visual input on the screen and count the number of times the task could be answered with “yes.” The shown stimulus always consisted of the elements necessary for all three external tasks and did not depend on the current task type (see Figure 1B). The trials lasted 10–14 s each and consisted of 8–11 stimulus screens of the same category. The trial length and type were chosen randomly. The stimulus screen (800 ms) was alternated with a masked screen (400 ms) between the single tasks. For example, for an external numerical trial, the task was to count how many times the shown number comparison was correct (i.e., 9 < 7). By always displaying a very similar visual stimulus, the differences between trials were minimized and restricted to the explicit task. Accordingly, the same presentation of visual stimulus screens was chosen for internal tasks even though their content was irrelevant for the tasks. An exemplary internal task was to generate as many words as possible starting with the letter D, without saying them out loud. Performance checks were randomly presented in 1/4 of the trials. A full data set of one participant consisted of two experiment blocks with 8 trials of each task in a randomized order (96 trials in total). Incomplete data sets were also included in our analysis.

For the binocular eye data recording an SMI RED250mobile system (SensoMotoric Instruments, Germany) with a temporal resolution of 250 Hz, spatial resolution of 0.03°, and gaze position accuracy of 0.4° visual angle was used. The participants' heads were stabilized using a chin rest.

#### 2.1.2. Alignment-Task

**Figure 2:**
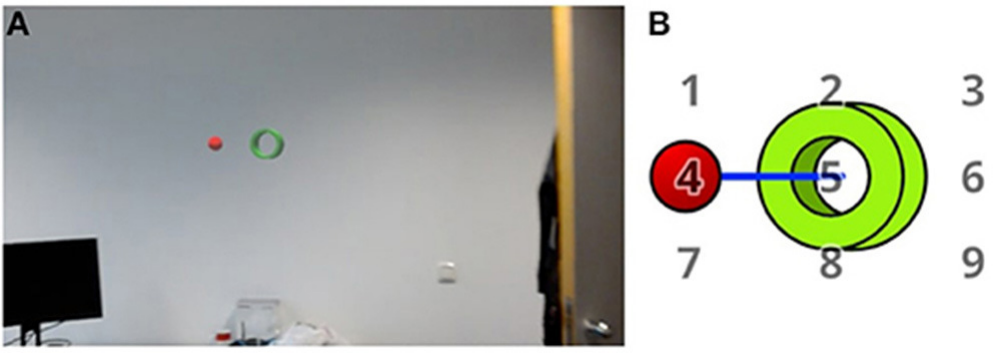
The alignment-task: **(A)** Example scene from the HoloLens, showing green tube and unaligned red ball in front of a white wall. **(B)** Schematic display of the tube and the ball together with the imagined number pad for internal trials. The blue line indicates a ball movement from 4 to 5. Taken from Vortmann et al. (2019a).

In Vortmann et al. (2019a), the alignment-task of the second data set was described. In this study, internally and externally directed attention was evoked during an Augmented Reality scenario. The task of the participants was to visually align a virtual ball (red) and a virtual tube (green) that can be seen in Figure 2A. During the trials with externally directed attention, the ball kept moving in slow steady motions with direction changes every 5 s within a small distance from the center of the tube to keep the participant focused for 20 s. The tube was in a fixed position while the ball moved on a plane that was parallel to the surface of the tube but closer to the participant than the tube. The alignment was achieved by movement of the upper body and head. For the trials of internally directed attention, the participants learned to imagine the movement pattern of the ball based on a series of numbers. In a tutorial, the ball and/or a number pad were displayed in front of the tube (see schematic representation in Figure 2B). In the real internal trials, this number pad and ball had to be imagined by the participant. Before such a trial, a sequence of 3 numbers between 1 and 9 was played as audio (i.e., 1-6-8). This sequence described the motion pattern of the imagined ball (i.e., upper left–middle right, lower middle). The participant's task was to imagine the movement and always slowly adjust their current position to keep the ball and tube aligned. They were advised to take approximately 5 s to imagine the movement of the ball from one number to the next number, resulting in a total trial time for internal trials of 15 s. Taken together, the task was always to keep the visual or imagined ball “inside” the tube by adjusting one's position. This task design was chosen to have two identical conditions regarding movement and visual input type while differing in the state of attention.

Participants performed 36 internal and 36 external alternating trials in total, split up into 3 blocks with breaks in between. The holograms and sounds were displayed using a Microsoft HoloLens 1. A binocular PupilLabs eye tracker with a sampling rate of 120 Hz was attached to the screen of the HoloLens to record the eye gaze. The average eye tracker accuracy is not available for this dataset.

#### 2.1.3. Pairs-Task

**Figure 3:**
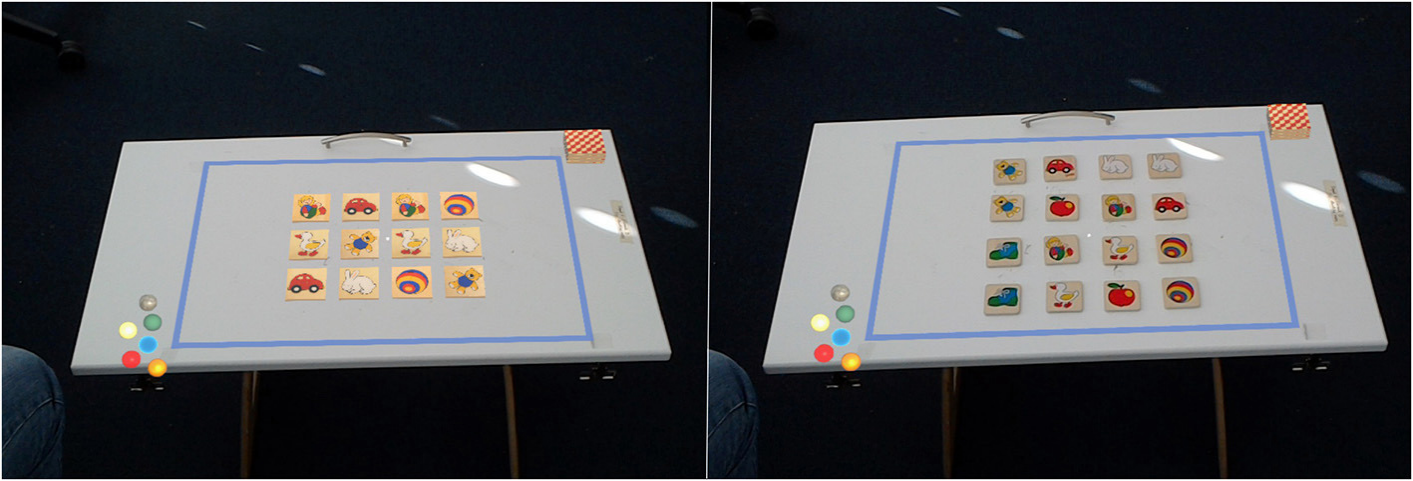
The pairs-task: screenshots from the HoloLens showing the setup of the game. Virtual marbles and a deck of cards are always visible. On the **left** image the pairs cards are virtual, on the **right** image the cards are real. Taken from Vortmann et al. (2021).

The third data set was recorded during the performance of a pairs-task that was described in Vortmann et al. (2021). For this experiment, the participants had to play the children's game “pairs” with two different conditions in Augmented Reality. During the game, the participants have to memorize the positions of several cards. Each picture is present twice. These two cards are a pair and have to be identified as such while the cards are turned over to their neutral side with no pictures on them. In the first condition, the cards are real wooden cards while some of the surrounding elements are augmented content. In the second condition, the same cards with similar symbols are virtually added to the scene (see Figure 3). During the “memory”-phase, the participants see a deck of cards with the picture side up for 20 s and have time to memorize as many of the pairs as possible (varying deck sizes for different difficulties). Afterward, in the “remember”-phase, the participants can choose the pairs that they remembered. For the classification task, only the “memory”-phase will be regarded. During these 20 s, it can be assumed that the participants exclusively pay attention to the real or virtual cards, depending on the condition. Because the task is exactly the same in both conditions, the same viewing strategy would be assumed. With this data set, the goal is to see whether it is possible to classify attention on real vs. on virtual objects in Augmented Reality settings based on eye tracking data.

The same setup of the HoloLens 1 and the PupilLabs eye tracker as in the alignment task was used in this setup. The participants performed 20 trials of each condition. Trials with technical problems were excluded from the analysis. The average eye tracking accuracy after the calibration was 2.49 ± 0.51 degrees and on average 0.4 trials were excluded.

### 2.2. Classification Algorithms

To classify the different trial conditions in the presented data sets, different features, feature sets, and classification algorithms can be combined to optimize the classification performance. The goal of this study is to improve attentional state classification accuracy based on eye tracking data by following a new Imaging Time Series approach for the feature extraction. We will first describe which features were extracted for the statistical summary approach that was inspired by state-of-the-art related studies and will be used as a benchmark to compare the new approach to. This 1D feature set will be used to train several different classification algorithms. The ITS approach will contain a feature matrix of several generated images that will be used to train two different convolutional neural networks, which we will describe in section 2.2.2. No further preprocessing was applied to any of the datasets and no trials were excluded, other than already mentioned in section 2.1.3.

#### 2.2.1. Statistical Summary Approaches

The general task-independent eye tracking features that are usually extracted were described in section 1.1. Which features can be extracted from the data sets is restricted by the format of the variables and values that were recorded by the eye trackers during the experiments. For some of the vergence features suggested by Xuelin Huang et al. (2019) information about the distance between the eyes and the distance between the focused object and the eyes is necessary. However, these are not given for all our data sets and thus we decided to combine the statistical summary feature set from fixations, saccades, blinks, remaining vergence features, and pupillometric data. For the extraction of these features, the data sequences of X and Y coordinates were evaluated for fixations, saccades, and blinks using the PyGaze Toolbox (Dalmaijer et al., 2014). The threshold value for the blink detection algorithm was 50 ms. Fixations were detected following the dispersion threshold identification algorithm (I-DT) by Salvucci and Goldberg (2000) (Implementation on github^1^). The dispersion threshold was set to 1 degree, as suggested by Blignaut (2009). The remaining vergence features were extracted as described in Xuelin Huang et al. (2019) and the minimal bounding circles were calculated with the python script from the nayuki-project^2^. As a feature, we either used the total value of the calculated variable, if possible (i.e., number of saccades), or calculated statistical measures to describe the variable during the trial (i.e., mean, standard deviation, median, maximum, minimum, range, kurtosis, and skewness of the distribution of saccade lengths). For a complete list of all 76 features see the Appendix.

After feature extraction, all features are normalized using a z-score normalization. Features are ranked using an ANOVA estimator and a non-parametric mutual information estimator. These feature selection approaches were implemented using the scikit-learn toolbox by Pedregosa et al. (2011). As a hyperparameter optimization, we used the 10, 20, 30, 40, 50, 60, and 70 highest ranked features of both estimators.

The classification **algorithms** were also implemented using the default implementations from scikit-learn. We implemented the pipeline with the following algorithms:

- Naïve Bayes (NB)
- Logistic Regression (LogReg)
- Random Forest (RF)
- k-Nearest-Neighbor (knn)
- Linear Support Vector Machine (linSVM)
- Multi Layer Perceptron (MLP)
- and AdaBoost

The best feature set was chosen for each classifier individually by computing the average classification accuracy of all folds during five-fold cross-validation. The whole pipeline can be seen in Figure 8 in the counter-clockwise path. This approach is used to gain optimal performance out of the classical approach, not considering any side-effects that could be caused by multiple testing of many classifier and feature set combinations (as they can only be beneficial for the classifiers and you are mainly interested in an upper bound).

#### 2.2.2. Imaging Time Series

For the ITS approach, the continuous X and Y coordinate variables were transformed into images and classified using a neural net. In a preliminary step, phases during which blinks were detected were filtered from the data, because no information about the X and Y coordinates is available. A detailed description of the methods can be found in Wang and Oates (2015).

**Figure 4:**
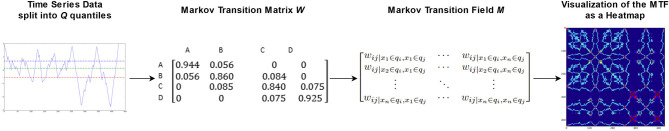
Flowchart representing the main steps of the MTF algorithm. Adapted from Wang and Oates (2015).

We decided to generate the images separately for the right and the left eye with one image representing the X coordinate and one image representing the Y coordinate recorded by the eye tracker. This way, we stay closest to visualizing the raw data and give the neural net the additional possibility to detect and learn from the differences and similarities between the eyes (following the idea of using vergence features). The first algorithm used for the transformation is the **Markov Transition Field** (MTF) which generates a matrix using transition probabilities. Based on the magnitude of the values, the data sequence *S* is split into *Q* quantiles. Each data point *x*_*i*_ is assigned to a quantile and a *Q* × *Q* weighed adjacency matrix *W* is constructed by counting the transitions from sample to sample between quantiles through a first-order Markov chain along the time axis. This Markov transition matrix *W* is then normalized and spread out among the magnitude axis considering the temporal positions, resulting in the MTF *M*. The main diagonal *M*_*ii*_ shows the self-transition probability at each time step (see Figure 4).

**Figure 5:**
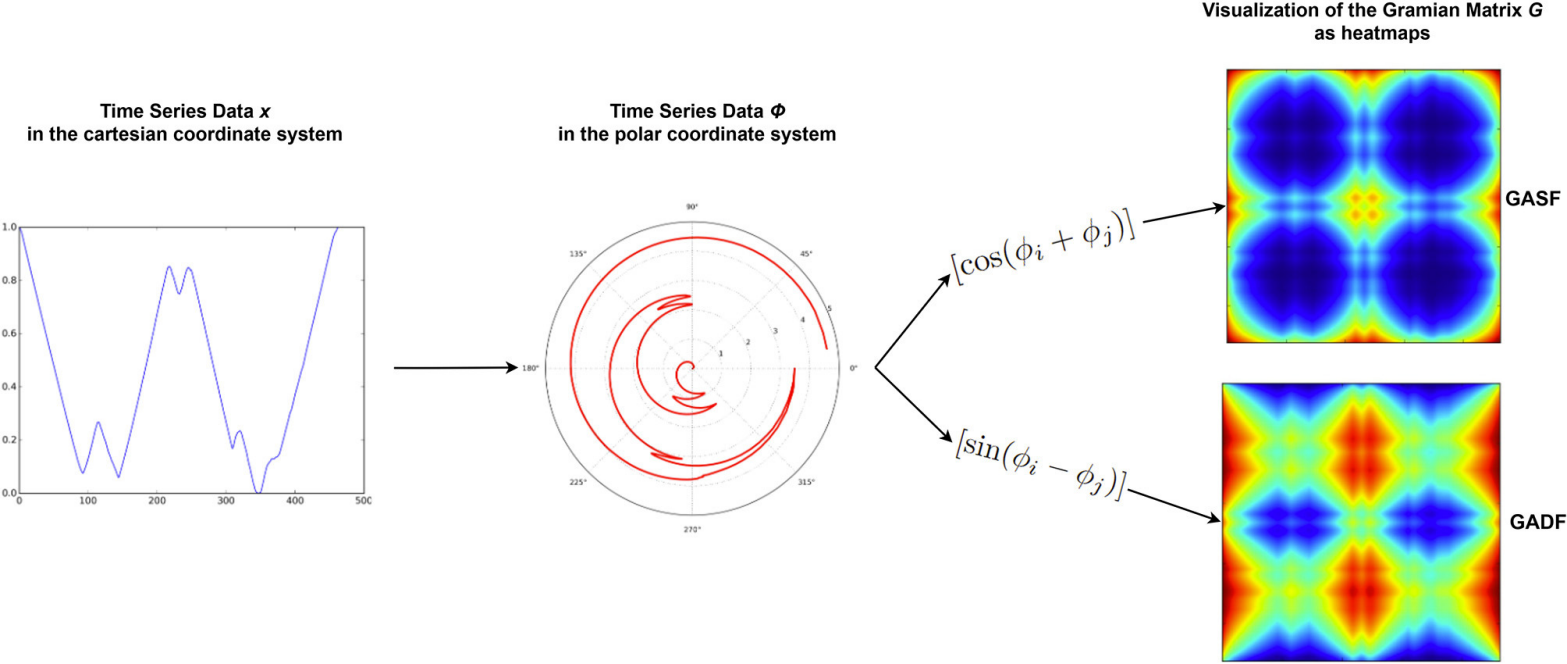
Flowchart representing the main steps of the GAF algorithm. Adapted from Wang and Oates (2015).

Additionally, we will work with two different versions of the Gramian Angular Field transformation algorithm. The first is called **Gramian Angular Summation Field** (GASF) and the second is called **Gramian Angular Difference Field** (GADF). For both methods, the data sequence *X* is rescaled to [−1, 1] and then represented in polar coordinates by encoding the data values *x* as the angular cosine and the according timestamp as the radius. Thus, the data sequence is transferred from the Cartesian coordinate system into the polar coordinate system which has the advantage that for all points we preserve the absolute temporal relation. In the final step, we calculate the trigonometric sum (using cosine for the GASF) or the trigonometric difference (using sine for the GADF) pairwise between the points to identify the temporal correlation within time intervals. Accordingly, the Gramian matrix *G* has a size of *n x n* with *n*= length of raw time series. Each cell *g*_*ij*_ of *G* represents the trigonometric difference/sum of the points *x*_*i*_ and *x*_*j*_ with respect to the time interval. On the main diagonal, each cell *g*_*ii*_ contains the original value/angular information and could be used to reconstruct the original time series *X*. The steps of this algorithm are visualized in Figure 5, where Φ represents the time series in polar coordinates.

To reduce the size of the generated images, Piecewise Aggregation Approximation (PAA) can be applied for blurring (Keogh and Pazzani, 2000). The effect of blurring will be discussed in section 3.1.1.

**Figure 6:**
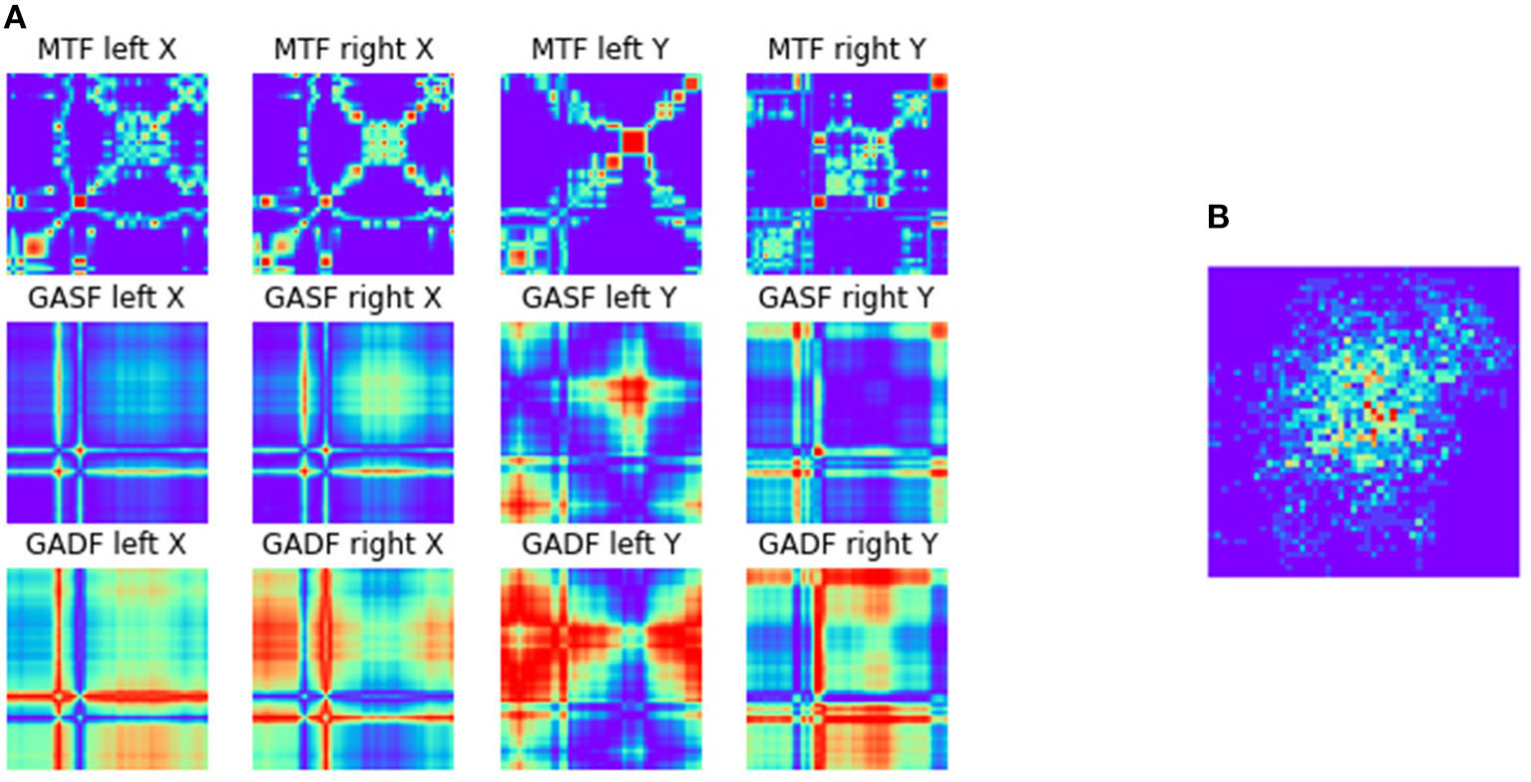
**(A)** Exemplary feature matrix (3 × 4) made up of 12 images generated during the ITS approach as described in section 2.2.2. An external numeric trial from the switch-task is represented. Each row represents one of the transformation algorithms with one image for each eye/axis combination. **(B)** Heatmap of the gaze points representing the same trial.

The transformations of the data sequences into the MTF, GASF, and GADF images were implemented using the pyts-toolbox for python (Faouzi and Janati, 2020). The image size was set to 48*x*48 pixels and all pixel values were normalized between [−1, 1] for individual images. Afterward, all generated images (3 transformations × 2 eyes × X/Y-coordinates = 12 images) were combined into an image matrix of size 3*x*4. This image generation process was applied to valid (non-blink) data of single trials per condition. An example of the images representing the feature matrix for an external trial of the switch-task data set can be seen in Figure 6A.

**Figure 7:**
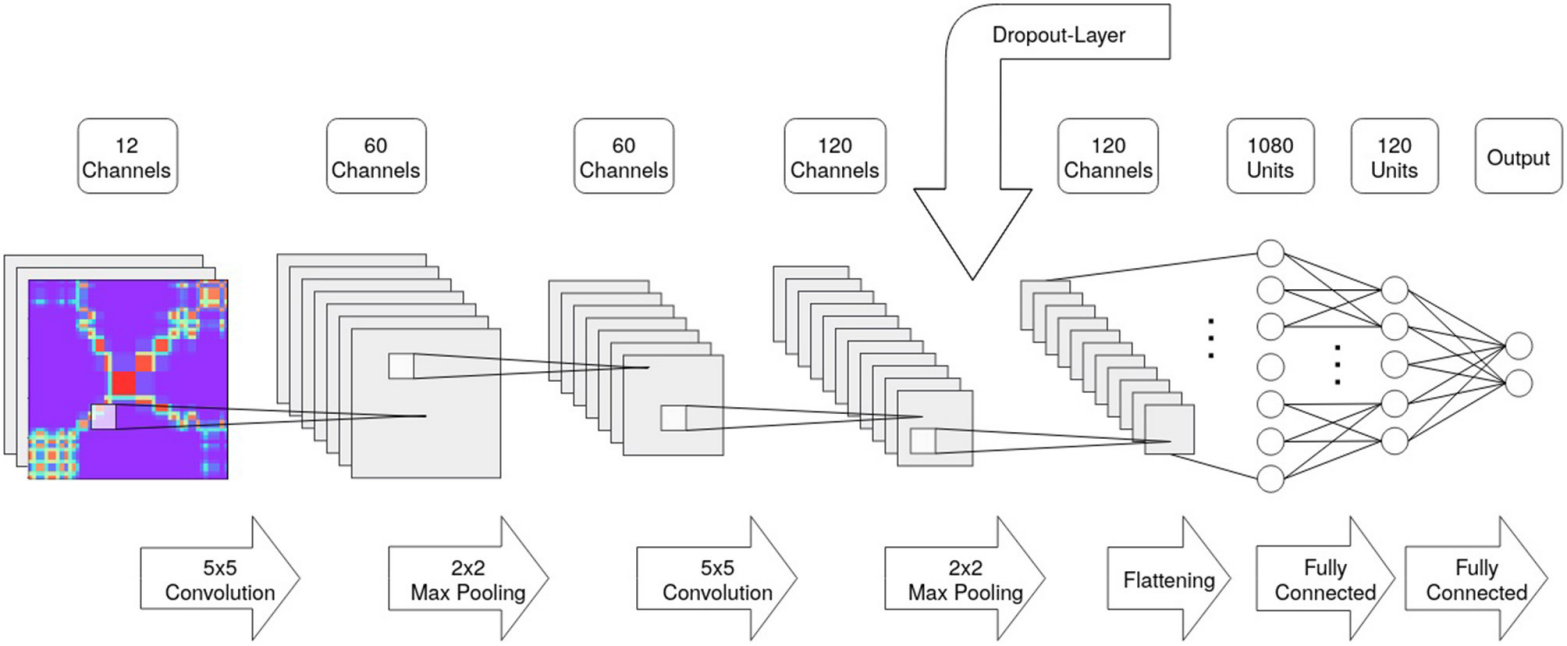
Schematic description of the SimpleNet structure indicating layer size and number of channels/units.

For the classification of the resulting images, we chose two CNNs with different complexities. The first CNN will be called **SimpleNet** and was implemented following the suggestions of Yang et al. (2020). It is made up of two convolutional layers with a kernel size of 5*x*5, two Max Pooling layers with a window size of 2*x*2 pixels, and two fully connected layers as well as the output layer. The number of units of the output layer is identical to the number of possible classification labels (in our cases: 2). Additionally, a dropout layer was included that temporarily freezes learned weights to avoid overfitting (see Figure 7 for a schematic representation of the SimpleNet).

The second CNN is the **AlexNet** (Krizhevsky et al., 2017) that won the ImageNet Large Scale Visual Recognition Competition in 2012 (trained from scratch). It is more complex than the SimpleNet as it consists of 5 convolutional, 3 max-pooling, and 3 fully connected layers that are initialized with more channels/units. The learnable parameters in the AlexNet (57,081,730) are 41 times as many as in the SimpleNet (1,364,942). As in the statistical summary approach, the CNNs were trained in a five-fold cross-validation.

### 2.3. Analysis

For the classification, all trials of one data set were cut to the same length to avoid that the classifiers learn length-related information instead of attention-related information. That means, all trials of the switch data set were cut off after 10 s (equal task contribution was given) and the alignment-task data was shortened to 15-s windows for both conditions. The trials in the pairs data set were all equal in length and were thus kept at 20 s.

**Figure 8:**
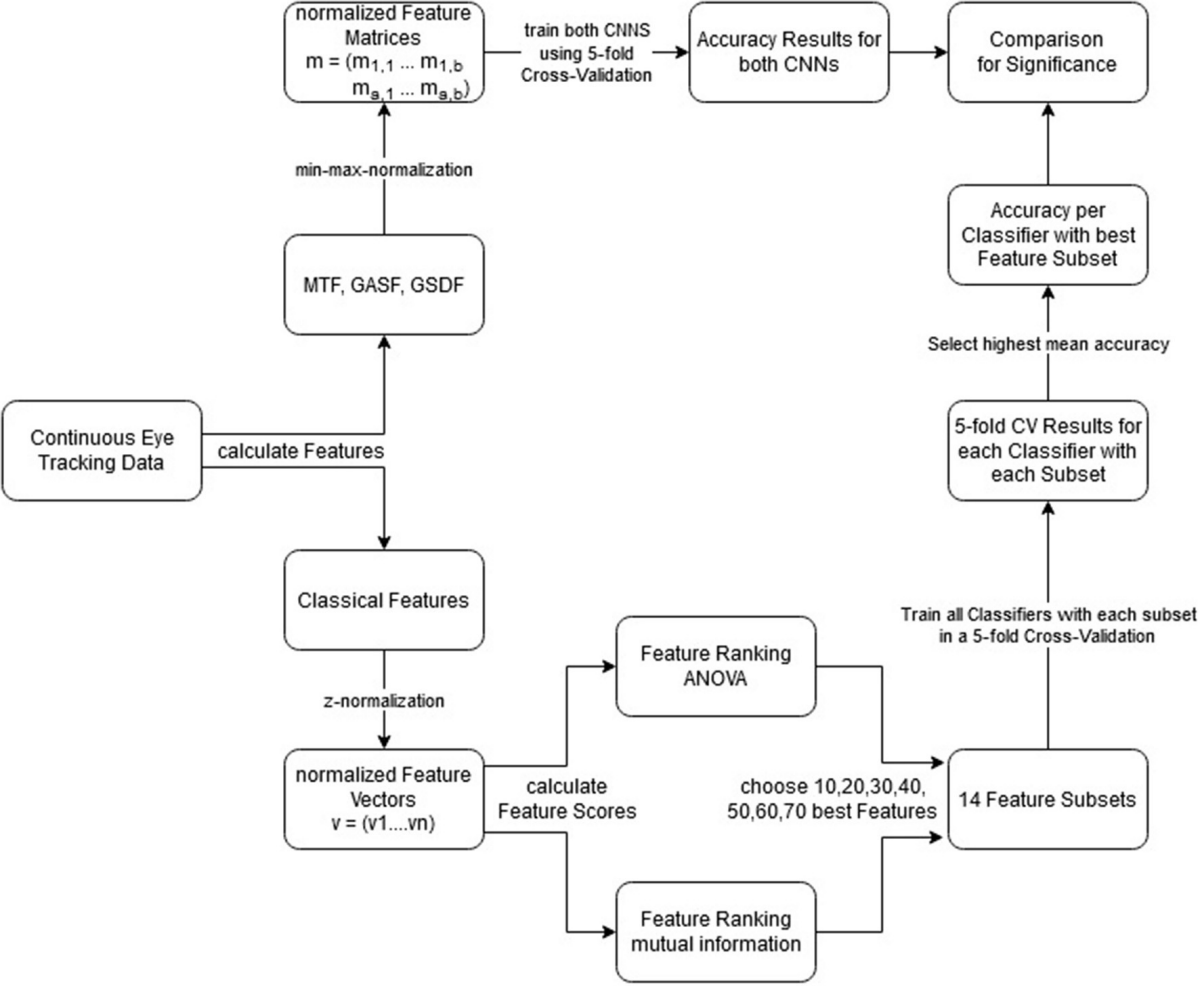
Combined pipelines of the statistical summary and the ITS approach. Explaining step by step what was done to get from the continuous eye tracking data to the performed comparisons. Clockwise, ITS approach; Counter-Clockwise, statistical summary approach.

The full pipeline—from the data sets to the comparison of the classifiers—can be seen in Figure 8. The counter-clockwise path shows the statistical summary approach and the clockwise path shows the ITS approach. As a performance metric, we chose to compare the resulting classification accuracies. This is possible because the attentional states were represented equally in the data sets. Accordingly, the chance level accuracy of guessing the correct attentional state for the binary classification tasks was 0.5.

For comparison of the classification accuracies, we want to determine whether one algorithm offers a statistically significant improvement over another approach. Therefore, we used a Wilcoxon-Signed Rank Test with a significance level of α = 0.05. Paired data sets were assured by using reproducible training-test-splits across classifiers. Since we want to test whether one algorithm is not just different, but actually better (in this case returning lower values) than the other algorithm, we use the one-tailed version.

In the following, all comparisons will be presented in tables displaying the p-values of the one-tailed Wilcoxon-Signed-Rank Test. If a *p* < 0.05 is reported, that means that the classifier in that row performed significantly better than the classifier in that column. All values were rounded to 3 decimal places, thus, values of 1 and 0 are possible (0 meaning highly significant improvement). It follows that, if there is no *p* < 0.05 in one row, the classifier in that row did not perform significantly better than any other classifier. A “better” performance means a more accurate classification. Additionally, we report the mean classification accuracies for all classifiers in the tables.

For the training and testing splits, we followed three different strategies to answer three different research questions regarding the generalizability of the data. First, we train and test individually on data from the same participant (person-dependent, section 2.3.1). Afterwards, we test how well the data generalizes over participants (person-independent, section 2.3.2) and over tasks (task-generalizability, section 2.3.3).

#### 2.3.1. Person-Dependent Classification

A person-dependent classifier is trained on data from one person and used to classify other data of the same person. For this approach, we took a participant's data from one dataset and performed a five-fold cross-validation with each of the suggested classification algorithms. For the statistical comparisons, the mean classification accuracy over the folds per participant was compared.

For reasons of computational time, only the SimpleNet was used with the ITS features during this analysis. The results are reported in section 3.2 and Table 2.

**Table 2:** Person-dependent: Average classification accuracies over all participants if the classifier was trained in a person-dependent manner; ***bold and italic***, highest average accuracy for this task; **bold**, *p*-value of the one-sided Wilcoxon Signed Rank Test above 0.05, thus no statistical difference between this and the best performing classifier.

	**simpNet**	**knn**	**linSVM**	**RF**	**MLP**	**AdaBoost**	**NB**	**LogReg**
Switch	***0.694***	0.609	0.619	0.58	0.571	0.559	0.612	0.604
Align	***0.707***	**0.667**	0.633	0.579	0.628	0.617	0.632	0.601
Pairs	***0.662***	0.589	**0.647**	0.585	0.614	0.524	**0.652**	0.582

#### 2.3.2. Person-Independent Classification

The person-independent version of the classifiers is trained on data that is independent of the participants whose data it is tested on. For this analysis, a combined data set over all participants per task is split and trained/tested using a group-five-fold cross-validation. That means the five-folds are chosen in a way that the data from one participant can never be in the training and in the testing data subset of that fold. The statistical comparisons are performed on the accuracy results of the individual folds.

The results are reported in section 3.3 and Table 3.

**Table 3:** Person-independent: Average classification accuracies over all folds of the group-five-fold cross-validation for the person-independent classifier; ***bold and italic***, highest accuracy for this task; **bold**, *p*-value of the one-sided Wilcoxon Signed Rank Test above 0.05, thus no statistical difference between this and the best performing classifier.

	**simpNet**	**alexNet**	**knn**	**linSVM**	**RF**	**MLP**	**AdaBoost**	**NB**	**LogReg**
Switch	***0.743***	0.73	0.642	0.685	0.674	0.688	0.69	0.619	0.689
Align	0.619	***0.705***	0.602	0.596	0.609	**0.641**	0.606	0.555	0.603
Pairs	0.52	0.5	**0.778**	**0.783**	**0.793**	**0.806**	**0.802**	0.715	***0.808***

#### 2.3.3. Task-Generalization

The switch-task data set contains an equal share of trials from 6 different tasks, 3 of which require internally directed attention and 3 of which require externally directed attention. As a final analysis, we wanted to test how the classifiers perform when they have to generalize over tasks. Analogously to the person-independent approach, we test the classifier on a task that it has not been trained on in a leave-one-out cross-validation (LOOCV). For example, we train the classifier using all trials, over all participants from the three external tasks and the numeric and verbal internal tasks but we test whether it correctly classifies all trials from the internal visuo-spatial task as internal. To do this, we chose a leave-one-task-out cross-validation. Again, the statistical analyses are performed on the accuracies of the folds.

The results can be seen in section 3.4 and Table 4.

**Table 4:** Switch-task results, task-generalization: Average classification accuracies over all participants if the classifier was trained in using a LOOCV for each task in the switch dataset; ***bold and italic***, highest average accuracy; **bold**, *p*-value of the one-sided Wilcoxon Signed Rank Test above 0.05, thus no statistical difference between this and the best performing classifier.

	**simpNet**	**alexNet**	**knn**	**linSVM**	**RF**	**MLP**	**AdaBoost**	**NB**	**LogReg**
LOOCV	***0.783***	0.764	0.663	0.69	0.681	0.707	0.7	0.62	0.693

## 3. Results

Before the final comparison of all classifier implementations as described in section 2.3, we performed some preliminary tests to verify our approach and test the configurations regarding the optimal resolution of the images for the ITS approach.

### 3.1. Preliminary Tests

As suggested by Wang and Oates (2015), a blurring kernel can be used to decrease the resolution of the resulting images of the MTF, GASF, and GADF transformations. We were interested in how far a smaller image would lessen the classification accuracy because smaller images would lead to a reduced computation time (see section 3.1.1). Additionally, aiming at explainable AI, we had a look at the learned filters of the CNNs to assess whether the learned information is comparable to what is learned during image classification of real-world objects and whether we can understand what the CNN learns (see section 3.1.2). To test the hypothesis that the classifiers learn something about the differences between the conditions simply from different placements of the tasks in the visual field, we also trained our SimpleNet using heatmaps of the gaze coordinates and compared the results to the ITS approach (see section 3.1.3).

#### 3.1.1. Image Resolution

To test the effect of the image resolution, we chose the same training and testing approach as described to the person-independent classifier (see section 2.3.2). We compared an image size of 12 × 12, 24 × 24, 36 × 36, and 48 × 48 pixels on the switch- and the alignment-task data sets as examples. Because the overall results of the pairs-data set were not significantly better than chance, we did not perform this comparison on this data set.

For both data sets, we find a better classification performance for a higher image resolution. For the switch-task data set, the classification accuracy improves significantly with a higher resolution up to a resolution of 36 × 36 pixels (*p* = 0.0156 compared to 24 × 24 pixels). Images with a resolution of 48 × 48 pixels lead to a higher mean accuracy with a lower variance, however, this improvement was not significant for our comparison.

For the alignment-task data set, the classification performance does not improve significantly for resolutions higher than 24 × 24 pixels. However, the mean accuracy still increases and the variance decreases with higher resolutions.

For the following analyses, we used an image resolution of 48 × 48 pixels because our computation time was of minor importance. However, if this approach is used in other studies, smaller image sizes can be chosen without significant performance loss.

#### 3.1.2. Feature Analysis

The main reasoning behind using images that represent information from the raw data is that the Neural Net can abstract features that would not have been represented by an explicitly defined feature set. However, this is often argued to be a black box approach because it only tells us that there is a difference in the data but not what that difference is. Learning from clearly defined feature sets often allows for a detailed analysis on the importance of single features and thus, which features contain information about the differences between the conditions.

**Figure 9:**
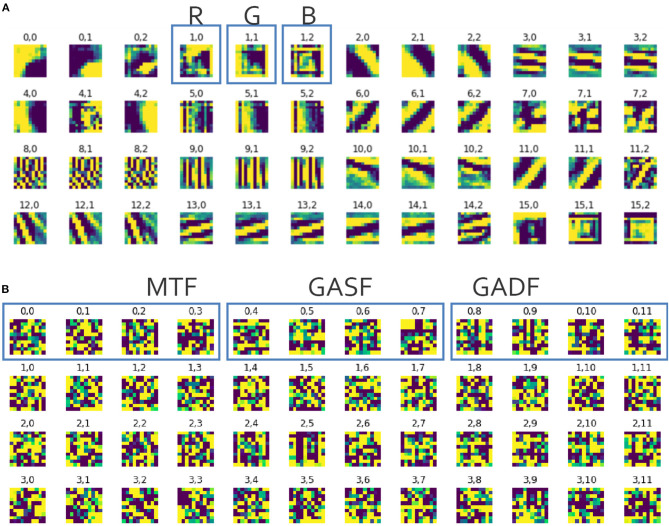
Excerpt from the visualization of the filters learned by the first convolutional layer of **(A)** the AlexNet trained on an image data set of real objects (i.e., animals and fruit) where X.0-X.2 represent the RGB-channels, and **(B)** the SimpleNet trained on ITS features, where X.0-X.3 represent the MTF images, X.4-X.7 represent the GASF images and X.8-X.11 represent the GADF images.

If a CNN is trained on images with real objects, the learned features often represent lines, edges, and other shapes (Krizhevsky et al., 2017). We visualized the features that were learned by the SimpleNet and found no such clear shapes or any other pattern that would explain what the CNN is learning from the ITS feature matrices (see Figure 9).

#### 3.1.3. Heatmap Analysis

To shine some light on the question of whether the CNN abstracts pure spatial information from the ITS features, we generated heat maps for all the trials of all data sets and compared the achieved classification accuracies for the person-independent approach using the SimpleNet. An exemplary heatmap can be seen in Figure 6B. For the alignment- and the pairs-task data set, the classification performance was not significantly different from chance level (0.5). For the switch task data set the classification reached an average over all folds of 0.631 which suggests that there is some spatial information in the data set that allows for a differentiation between the internal and external condition. These results will be discussed further in section 4.

### 3.2. Person-Dependence

For the switch-task data set, the person-dependent classifiers were trained on approximately 70 trials in each fold. The highest mean classification accuracy of 69.4% across all participants was reached by the ITS-SimpleNet classifier. This result is significantly better than all statistical summary approaches. The second-best classification result was achieved by the linear Support Vector Machine (SVM) classifier with 61.9%.

The training subsets for the alignment task contained approximately 55 trials. Again, the highest classification accuracy was reached by the SimpleNet with 70.7% correctly classified trials on average. In this case, it was not significantly better than the best performing statistical summary classifier, which was the k-Nearest Neighbors approach with 66.7%. The SimpleNet is significantly better than all other tested classifiers.

In the pairs-task data set, the training subset for the person-dependent classifiers includes approximately 30 trials. As for the other two data sets, the highest classification accuracy is reported for the SimpleNet (66.2%) but with no significant improvement compared to the Naïve Bayes algorithm (65.2%) and the linear SVM (64.7%) (see Table 2).

Taken together, the SimpleNet reached the highest average accuracy for all three data sets if tested person-dependently with a significant improvement over all other statistical summary classifiers for the switch-task.

### 3.3. Person-Independence

Due to the combined data of the participants, the training subsets of the switch-task data set comprised approximately 12,000 trials for every fold in the person-independent approach. The classifiers that were trained on the ITS feature set performed significantly better than any of the statistical summary classifiers. The SimpleNet outperformed the AlexNet significantly with an accuracy of 74.3% compared to 73%. Of the statistical summary approaches, the linear SVM, the Random Forest, the Multi Layer Perceptron, the AdaBoost, and the Logistic Regression all classified approximately 68% of the trials correctly with no significant improvement over each other.

For the alignment data set, the combined trials result in training subsets of approximately 720 trials. The AlexNet had the highest classification accuracy of 70.5% on average over the folds. Only the Multi Layer Perceptron was not significantly worse with an accuracy of 64%.

The person-independent data set of the pairs-task resulted in approximately 320 training trials for each fold. The statistical summary approaches—except for the Naïve Bayes—reached accuracies of up to 80% with no significant statistical improvements over each other. The SimpleNet and the AlexNet only reached accuracies around 50% which is comparable to guessing (see Table 3).

The results show, that it is possible for all three data sets to generalize over all participants. However, which feature set captures the differences and similarities best is highly dependent on the attentional states that are to be classified.

### 3.4. Task-Generalizability

For the last analysis, the task independence of the features was tested by combining the switch-task trials of all participants and testing on only one of the six tasks. This resulted in approximately 12,500 trials in the training set. The best classification accuracy was achieved using the SimpleNet. It classified on average 78.3% of the trials correctly as internal or external attention even though it had never learned on trials from that task. This was significantly better than all the other classifiers. The second best classifier was the AlexNet with an accuracy of 76.4% which was significantly better than all statistical summary approaches. The best statistical summary approaches were the Multi Layer Perceptron, AdaBoost, and the Logistic Regression with up to 70.9% (see Table 4).

## 4. Discussion

To optimize the accuracy of attentional state classification based on eye tracking data, different methods of feature extraction for various feature sets in combination with several classifiers have been tested in the past. In this work, we followed a new path by using an Imaging Time Series approach to visualize the raw eye tracking data and to classify the resulting images using convolutional neural networks. We compared the results with classical state-of-the-art approaches and found that our ITS approach outperforms the other classifiers. This difference can not be an advantage of deep learning in general, because the Multi Layer Perceptron that was trained on the statistical summary feature set was also significantly worse than the ITS approaches. However, a comparison between different image generation algorithms as features for the same deep learning classifier has yet to be assessed.

Even though the smallest amount of training data was used for person-dependently trained classifiers, the CNNs outperformed the general feature set classifiers in all three data sets. Interestingly, for the pairs data set, the CNNs that were trained person-independently on the ITS features did not achieve accuracies better than chance level, despite the bigger training data set. Since the classification was significantly better for the person-dependent classification, we assume that the ITS approach captures some characteristics of the eye gaze behavior that are different between the attention on real and virtual objects. However, the bad person-independent results suggest that the information that is captured in the ITS features is very individual between participants regarding viewing behavior. The statistical summary features and classifiers reached accuracies up to 80% for this task, thus, there are person-independent eye gaze feature differences during attention on real and virtual objects, these are just not learned in the ITS approach. Understanding this result requires further insight into the information that is encoded into the images and which filters were learned by the convolutional neural net. So far, the only conclusion we can draw from this is that the statistical features contain information that is missing in the ITS approach but would be important to classify attention on real and virtual objects in a person-independent manner. We excluded poorly randomized training and testing data as the reason for the low classification accuracy by using the same splits across classification approaches. Also, the comparatively small amount of available data has a low probability of causing the low performance because the person-dependent classification for the pairs task was performed on even fewer data and reached a better performance.

For the two internal/external data sets the highest accuracy for the approach that generalizes over participants was again reached by one of the suggested new classification approaches over the statistical summary approaches. What can be noted is that in the switch data set, the SimpleNet performs significantly better than the AlexNet, while for the alignment data set it is the other way around. The results between the two CNNs are similar for the pairs- and the switch-task (< 2%) but the accuracy for the SimpleNet used on the alignment data set is almost 9% worse than the AlexNet.

An interesting question that could be followed here is in how far the different complexities of the two models require different amounts of training data to reach similar results. The effect of more training data for CNNs was also discussed in Zhu et al. (2016) where they investigate the saturation threshold for the models. They conclude that while bigger data sets are almost always better, the real improvement happens when the representations of the data and the learning algorithms improve and are capable of profiting from larger data sets. While, a more complex model with more learnable parameters is more prone to overfitting if the amount of data is too small, it is also capable of capturing more complex structures. However, adding parameter complexity beyond the optimum reduces model quality. More training data is desirable because it reduces the variance in the model and displays more accurately which aspects of the data are general and which are the noise of specific trials. In our current analysis, we have not yet identified which characteristics of the two compared CNNs are responsible for the differences in the achieved classification accuracies. We assume, that the required complexity of the model is dependent on the attentional or in general mental states that are to be classified. This topic will need further investigation.

A very noticeable achievement is that the classification accuracies with the ITS approach for internal and external attention do not decrease for person-independent classification (74.3 and 70.2%) compared to person-dependent classification (69.4 and 70.7%) and for the pairs dataset it even increased (80.8% compared to 66.2%) when the Logistic Regression was chosen. For user applications that make real-time use of the classification results, a person-independent classifier eliminates the need for a long session of recordings just to train the classifier. This helps to develop real-time training-free use case scenarios where eye tracking data can be used to detect internally and externally directed attention in the user and if the attention is directed externally in Augmented reality settings, it can be classified whether the focus lies on real or virtual objects.

Another promising result is the high accuracy achieved for the task generalizability analysis. Using the ITS features together with the SimpleNet resulted in 78.3% correctly classified trials on average even though the classifier was not trained on data from that task. In Annerer-Walcher et al. (2021), the authors reported an accuracy of approximately 61% for their task transfer classification approach using an LSTM with the standard features. One difference is that they trained on two internal and two external tasks and tested on the remaining two. However, the classification accuracy reached by our approach is remarkably higher and we assume that not all of this difference can be explained by the different test/training split. We propose that the characteristics of the gaze behavior that are represented in the Imaging Time Series features are a good representation of what is shared over tasks during certain attentional states.

The trial lengths that were analyzed in this study (10–20 s) were adopted from the original studies for better comparability. To use the proposed methods in an online real-time system or for a temporally detailed offline classification, the approach should be adapted to either use smaller windows or sliding windows. While, smaller windows also reduce the available data for each decision, this is not the case for overlapping sliding windows. Appropriate window lengths or window overlaps for sliding windows highly depend on the context. While, some research questions might require a fine-grained analysis of attention switches (e.g., to study the exact steps of a single cognitive process), most applications would rather benefit from the detection of robust attention changes for longer periods (e.g., adapting a user interface to the attentional state, where too frequent changes would be more distracting than helpful).

Our study was the first to assess this classification approach for attentional states based on eye tracking. We were able to show an improvement in classification accuracy and are optimistic that further optimization can be achieved. A shortcoming of the presented analysis is that all the implemented classifiers were implemented in their default settings. Our goal was to use the same classifiers on all data sets and thus not optimize each classifier independently for each data set and classifier training variant. We are aware that the classification accuracy of the statistical summary approaches could be increased by performing further hyperparameter optimization additionally to the feature selection criteria. On the other hand, the CNNs that were used to classify the ITS features were also taken “out of the box” and were not optimized and designed specifically for this analysis. Typically, neural nets require a large amount of training data, which could be assessed in further experiments. We conclude that their results could be improved in the same dimensions that the statistical summary algorithms could be improved. Our goal was to show that this feature set is an interesting alternative that requires further attention because it might lead to better classifier performances.

A bigger challenge for the new approach is the interpretation of the model. While, the feature importance and differences can easily be analyzed for the statistical summary features, the parameters that are learned during the training of the CNNs with the images are harder to interpret. A pitfall of the ITS approach is its dependency on the gaze coordinates if these are the main difference for the learned conditions in the training data set. In the switch-task there seem to be differences between the conditions regarding the gaze heatmaps. A classifier should not learn that internally directed attention is present whenever the participants look to the left and externally directed attention is present whenever the participants look to the right because it is not task and location independent. The statistical summary features do not fall for this information. In our case, the results of the person-independent ITS classification (74.3%) are significantly better than the results using a heatmap of the gaze coordinates (63.1%) which shows that the classifier learns significantly more from the Imaging Time Series than the “location.”

All in all, the results of this first exploration of Imaging Time Series for eye tracking classification show that it is promising to further test and optimize in this direction, exploring other feature extraction and combination methods.

### 4.1. Future Work

In this work, the Imaging Time Series approach was tested on three different datasets. In the next step, other available eye tracking data sets of attentional states will be classified using this feature set. If possible, these data sets should contain other tasks and attentional states. The analyses will focus on understanding and optimizing the necessary complexity of the CNNs while keeping task- and person-independence in mind as a central goal.

After comparing the ITS approach to classical statistical gaze features, future comparisons will focus on other deep learning approaches that have been used on eye tracking data by related studies. In particular, we would be interested in a comparison of our suggested ITS approach with the approach from Sims and Conati (2020) where the CNNs were trained on the scanpaths and the temporal dimension was analyzed using GRUs.

Further, we want to investigate how well a combination of the statistical summary features and the ITS techniques mix. The statistical summary features contain a lot of information that is well-understood and can be explained by results from cognitive science research. However, with the statistical summary feature extraction and generation algorithms, a lot of information about the data is lost, especially with regard to the temporal dynamics within a trial. One idea would be to visualize some of the statistical summary features using Imaging Time Series. For example, the statistical summary features that describe the length of the saccades within a trial are often represented by statistical values that describe their distribution: Mean, standard deviation, minimum, and maximum. The saccade lengths are also a time series that could be transformed into an image with less information loss than the descriptive statistics. This could be an efficient combination of both approaches.

One last topic that was not addressed until now in this study is the window length of the classified data. With follow-up studies, we want to examine which effect the chosen time interval has on the classification accuracy. Precisely, shorter windows are desired if the accuracy loss is not significant because shorter trials would allow attentional state detection closer to real-time.

The overall goal will be an end-to-end system that can classify multiple aspects of the attentional state of a user without person-dependent training as fast and accurate as possible and use the information for adaptations of the interface or as implicit input.

## Data Availability Statement

The data analyzed in this study are available by request. Requests to access these datasets should be directed to Lisa-Marie Vortmann, vortmann@uni-bremen.de.

## Ethics Statement

The studies involving human participants were reviewed and approved by Ethics Committee, University of Bremen. The patients/participants provided their written informed consent to participate in this study.

## Author Contributions

The study was planned by L-MV, JK, and FP. The implementation was performed by JK. L-MV and JK analyzed and discussed the results. L-MV wrote the paper. SA-W, MB, and FP reviewed the paper. FP supervised the process. All authors contributed to the article and approved the submitted version.

## Conflict of Interest

The authors declare that the research was conducted in the absence of any commercial or financial relationships that could be construed as a potential conflict of interest.

## Footnotes

^1^https://github.com/ecekt/eyegaze (assessed December, 2020).

^2^https://www.nayuki.io/page/smallest-enclosing-circle (assessed December, 2020).

## Appendix

Complete list of features for the statistical summary feature set. sd, standard deviation; min, minimum; max, maximum.

- Distance between gaze points of both eyes mean
- Distance between gaze points of both eyes sd
- Angle between gaze points of both eyes mean
- Angle between gaze points of both eyes sd
- Distance between centroids of both eyes
- Angle between centroids of both eyes
- Distance between minimal bounding circles of both eyes
- Angle between minimal bounding circles of both eyes
- Normalized distance between minimal bounding circles of both eyes
- Minimal bounding circle radius left eye
- Minimal bounding circle radius right eye
- Fixation duration mean
- Fixation duration sd
- Fixation duration median
- Fixation duration min
- Fixation duration max
- Fixation duration range
- Fixation duration kurtosis
- Fixation duration skewness
- Fixation quantity
- Fixations total duration
- Saccade duration mean
- Saccade duration sd
- Saccade duration median
- Saccade duration min
- Saccade duration max
- Saccade duration range
- Saccade duration kurtosis
- Saccade duration skewness
- Saccade length mean
- Saccade length sd
- Saccade length median
- Saccade length min
- Saccade length max
- Saccade length range
- Saccade length kurtosis
- Saccade length skewness
- Saccade velocity mean
- Saccade velocity sd
- Saccade velocity median
- Saccade velocity min
- Saccade velocity max
- Saccade velocity range
- Saccade velocity kurtosis
- Saccade velocity skewness
- Saccade quantity
- Saccades total duration
- Angles between saccade and x-axis mean
- Angles between saccade and x-axis sd
- Angles between saccade and x-axis median
- Angles between saccade and x-axis min
- Angles between saccade and x-axis max
- Angles between saccade and x-axis range
- Angles between saccade and x-axis kurtosis
- Angles between saccade and x-axis skewness
- Angles between saccades mean
- Angles between saccades sd
- Angles between saccades median
- Angles between saccades min
- Angles between saccades max
- Angles between saccades range
- Angles between saccades kurtosis
- Angles between saccades skewness
- Fixation/saccade duration ratio
- Blink duration mean
- Blink duration sd
- Blink quantity
- Blinks total duration
- Pupil diameter mean
- Pupil diameter sd
- Pupil diameter median
- Pupil diameter min
- Pupil diameter max
- Pupil diameter range
- Pupil diameter kurtosis
- Pupil diameter skewness
